# General practitioners' explanation and advice on childhood eczema and factors influencing their treatment strategy: A qualitative study

**DOI:** 10.1002/ski2.147

**Published:** 2022-07-06

**Authors:** Karlijn F. van Halewijn, Tessa Warendorff, Arthur M. Bohnen, Mario Veen, Suzanne G. M. A. Pasmans, Patrick J. E. Bindels, Gijs Elshout

**Affiliations:** ^1^ Department of General Practice Erasmus MC, University Medical Center, Rotterdam Rotterdam The Netherlands; ^2^ Department of Dermatology, Center of Pediatric Dermatology Erasmus MC, University Medical Center, Rotterdam Rotterdam The Netherlands

## Abstract

**Background:**

Atopic dermatitis (AD) is common in children and the majority of children can be treated by the general practitioner (GP). Various factors can influence the GP's treatment strategy and may lead to less effective treatment. The objective is to gain insight into the treatment goal, treatment strategy, explanation and advice given by GPs when dealing with AD in children and to explore which factors play a role in the choice of pharmacological treatment.

**Methods:**

Semi‐structured interviews in primary care in the Netherlands were audio‐recorded and transcribed. All data were analysed according to the six‐steps approach of inductive thematic analysis.

**Results:**

We interviewed 16 GPs. Treatment goals mainly focussed on the short term. GPs discussed the importance of emollient use and emphasised emollients as the basis of treatment. We found that several factors played a role in prescribing topical corticosteroids (TCS); severity of the AD, age of the child, skin type, corticophobia among parents and GPs, experience of side effects and dermatological experience. GPs reported giving limited advice about the use of TCS and prescribed medication that is not recommended by the guideline.

**Conclusion:**

Various factors seem to influence GPs' treatment strategy for AD in children. More attention and education about the use and safety of TCS in children during GP training, continuous medical education, probably improve treatment in line with guidelines and can lead to more confidence and knowledge about TCS among GPs, which ultimately may improve the education and self‐management of patients.

## Graphical abstract

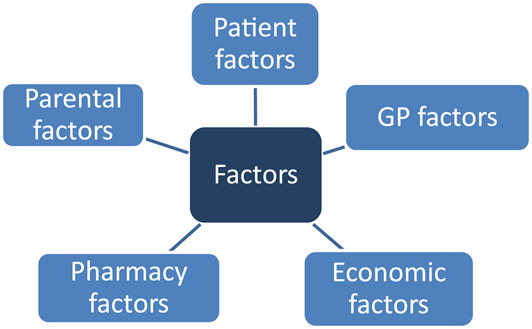

1

**What's already known about this topic?**

- The majority (70%–80%) of children with atopic dermatitis (AD) have mild disease which can be managed by the general practitioner (GP).
- Previous literature found that general practitioners are afraid to use potent topical corticosteroids (TCS) in children.

**What does this study add?**

- General practitioners reported that their main treatment goal was on the short‐term to get the children symptom‐free.
- This study found that general practitioners reported to give extensive general advice and put an emphasis on emollients as the basis of treatment.
- Divergent patient, parent, GP and pharmacy factors can affect the optimal treatment of AD in children.

**What are the clinical implications of this work?**

- General practitioners should put more emphasis on the long‐term goal in the treatment of AD.
- More education about the use and safety of TCS in children during the GP training and better implementation of the guideline is needed.
- Pharmacists (assistants) should also be educated about the use and safety of TCS in children to ensure that consistent information is spread by the primary care team.

## 2 INTRODUCTION

Atopic dermatitis, or eczema, is a common chronic disease in children. The prevalence of doctor diagnosed AD in children was reviewed by Bylund et al. and ranged from 1.8% to 22.6%.^1^ The majority (70%–80%) of children with AD have a mild ‐moderate disease which can be managed by the GP.^2^, ^3^, ^4^

The cornerstone of all AD treatment is emollients, usually in combination with TCS. Guidelines may differ, especially between primary and secondary care, regarding treatment strategies with TCS for paediatric AD. The Italian guideline advise starting with high‐potency TCS when AD flares up.^5^ The GP guideline of the Netherlands, and general guidelines of Scotland and Germany advocate a stepwise approach to treating a moderate flare‐up (Three Item Severity score 3–5): start with a mild TCS and if this is ineffective a moderate TCS or possibly a potent TCS, can be used. Very potent TCS are not recommended for treating AD in primary care in the Dutch GP‐guideline.^6^, ^7^, ^8^, ^9^ Also, GPs are supposed to provide the parents and/or child with general information about AD, for instance, about showering and bathing.

Despite the presence of national and international guidelines, many children with AD are underusing topical treatments and suffer more from their eczema than necessary,^10^ limiting their quality of life.^11^, ^12^ The use of emollients and topical corticosteroids is not always in line with AD guidelines.^10^ Divergent views between parents and GPs regarding disease aetiology, assessment and treatment of AD, in combination with parental concerns about TCS, can lead to less effective treatment of AD in children. Furthermore, previous literature found that GPs are afraid to use potent TCS in children.^13^ Also, corticophobia plays a role among parents and is caused by information found at the pharmacy, on the internet, or obtained from relatives or friends.^13^, ^14^, ^15^

In order to improve the treatment of AD in children, it is crucial to know how GPs manage AD and to understand their beliefs in relation to their medical treatment strategy. This qualitative study aims to explore what the treatment goal is and what information GPs provide to parents of children with AD. Furthermore, we want to examine what recommendations are given about emollients, bathing and other treatments in daily practice. Finally, we want to explore which factors play a role in the decision whether to prescribe TCS for treatment of AD in children. What information is provided to parents, and when do GPs consider prescribing a potent TCS?

## 3 METHODS

### 3.1 Setting and participants

The study was carried out among GPs in two regions in the southern and western parts of the Netherlands. Through an informative email 140 GPs were approached, in total 20 GPs responded positively. We used purposive sampling based on years of experience, prior dermatological experience was taken into account, including general working experience in dermatology and a dermatology internship during the GP training. In addition, GPs were asked whether they had been diagnosed with AD themselves and/or had a relative with AD.

### 3.2 Ethical considerations

All GPs received written information and provided written informed consent. Data were analysed and stored anonymously. The study was approved by the Medical Ethics committee of the Erasmus Medical Center, Rotterdam (MEC‐2019‐0626).

### 3.3 Data collection

Interviews were semi‐structured with open‐ended questions. We prepared a list of topics and composed questions based on the aims of the study and the available research literature, see Appendix 1. The topic guide was adjusted several times throughout the study using a continuous iterative process.

Data were collected in September and October 2019. The interviews took place in the Erasmus MC or in the GP practices and lasted between 15 and 40 min. Each interview was conducted by one researcher (Karlijn F. van Halewijn or Tessa Warendorff), and a second researcher (Tessa Warendorff or Karlijn F. van Halewijn) attended the interview. Tessa Warendorff was a master student and conducted interviews as a part of her master thesis, she was supervised during the interviews by Karlijn F. van Halewijn when Tessa Warendorff was the first interviewer. The interviews were audio recorded, anonymised, transcribed verbatim (Tessa Warendorff) and checked by a second researcher (Karlijn F. van Halewijn).

### 3.4 Analysis

We used inductive Thematic Analysis to analyse all transcripts, using the six analytic steps described by Braun and Clark.^16^ Two researches (Karlijn F. van Halewijn and Tessa Warendorff) individually coded two transcripts by hand and discussed them with a third researcher (Mario Veen). Because Tessa Warendorff was a master student we asked a third researcher to supervise the codes of the first two transcripts When agreement was reached on the codes, the transcripts were entered in the data analysis programme MAXQDA Analytics Pro and codes were given independently to two more transcripts. Consensus was reached through discussion and one investigator (Tessa Warendorff) then coded all the other transcripts since agreement was reached after four transcripts. All codes were compared both within and across interviews until the main themes and subthemes were developed (Tessa Warendorff and Karlijn F. van Halewijn). The main themes and subthemes were discussed within the wider research team (Tessa Warendorff, Karlijn F. van Halewijn, Mario Veen, Patrick J. E. Bindels, Arthur M. Bohnen, Suzanne G. M. A. Pasmans and Gijs Elshout) until consensus was achieved. See Appendix 2 for an overview of the final themes and subthemes.

### 3.5 Trustworthiness

Maximum variation in the data sample was achieved by including GPs with different numbers of years of experience, different practice locations and varying levels of dermatology experience. Diversity of perspectives in the research team was achieved by involving researchers from different backgrounds, that is, general practice (Karlijn F. van Halewijn, Tessa Warendorff, Patrick J. E. Bindels, Arthur M. Bohnen, Gijs Elshout), dermatology (Suzanne G. M. A. Pasmans) and communication and psychology (Mario Veen).

## 4 RESULTS

### 4.1 General

#### 4.1.1 General practitioner characteristics (Table 1)

**TABLE 1:** Characteristics of participants (*n* = 16)

Participants characteristics	Description
Mean years in role, years (range)	16.3 (0.25–38)
GP Partner or salaried	7 female, 6 male
Locum GP	3 female
Specialist interest in dermatology	1 female, 3 male
Personal experience of AD themselves	2 female
Personal experience of AD family member	2 female, 3 male

Abbreviations: AD, atopic dermatitis; GP, general practitioner.

In total 16 GPs were interviewed. Ten GPs were female (62.5%) and the years of experience as a GP ranged from 3 months to 38 years. There were four GPs with prior dermatological experience, two GPs had been diagnosed with AD or had had AD as a child and five GPs had a child with AD.

#### 4.1.2 GPs' treatment goals concerning atopic dermatitis in children

GPs reported that their main short‐term treatment goal was to get the children symptom‐free with no more itching or pain and restore disturbed sleep. Furthermore, they named fewer exacerbations and adequate self‐management as treatment goals.

#### 4.1.3 Information given about atopic dermatitis

When explaining the course of AD, GPs generally emphasised the chronic character of AD and the fact that it flares up. On the other hand, they offered the perspective that in most children AD can improve and disappear as they grow older. GPs stressed that treating AD requires a lot of attention from the parents.

Quote 1 (GP 6):

GP: “Well, then you explain that it can take a long time to go away and it’s something you have that can flare up from time to time and that it can also disappear for a while and, um… well, that it requires a lot of attention from the parents to treat it properly.”

#### 4.1.4 General treatment recommendations on emollients and bathing

GPs highlighted the use of emollients as the basis of treatment and they mentioned that the emollients should be used generously and as frequently as necessary. This in concordance with the Dutch GP‐guideline.

Quote 2 (GP 9):

And then I always start by telling them that the basis for eczema treatment is to keep the skin greasy. And I explain that dry skin is much more likely to get eczema patches, so keeping the skin a bit greasy is the key thing.

It was stated GPs indicated that they preferred ointment in the case of a very dry skin and cream in the case of oozing skin. Other GPs reported general preference concerning ointment or cream. Some GPs reported prescribing an ointment because it is the best choice because of the dry skin in AD, and it is more user‐friendly as it will stick to the skin. Other GPs reported to prescribe a cream because it spreads better and absorbs faster. Some had positive experience with using emollients for themselves or relatives, which influenced their prescription behaviour.

The advice to use emollients at least twice a day and more if needed was given by all GPs. Only one GP mentioned covering the whole body with emollients. None of the GPs gave advice about the amount of emollient to use.

GPs reported giving advice about bathing, showering and the use of soap in line with the Dutch GP guideline for AD. This recommends short (target time 5 min), lukewarm (37°C) and infrequent bathing/showering (children <1 year, 2–3 times a week) and use of only a little soap.^6^ Also the use of bath oil is broadly recommended by the GPs, which is also suggested by the Dutch GP‐guideline.^6^

It was remarked GPs referred patients to a certain Dutch website (www.thuisarts.nl) specifically designed for patients and containing reliable information provided by the Dutch College of General Practitioners (NHG).

#### 4.1.5 Other treatments

More than a third of GPs prescribed oral antihistamines if children were suffering from itching and sleep deprivation. Antihistamines are only justified under specific conditions, and are contraindicated for children under 2 years of age.^6^ Some GPs reported prescribing tacrolimus (local calcineurin inhibitor) for sensitive areas like the eyelids. The use of local calcineurin inhibitors to treat eczema in primary care is not recommended by the Dutch GP‐guideline.^6^ Only two GPs mentioned that they prescribed therapeutic clothing (i.e. silk clothing) if a potent TCS was not sufficient in children with severe AD. Therapeutic clothing can be considered for extensive moderate or severe atopic eczema as suggested by the Dutch GP‐guideline.^6^

### 4.2 Topical corticosteroids

**FIGURE 1:**
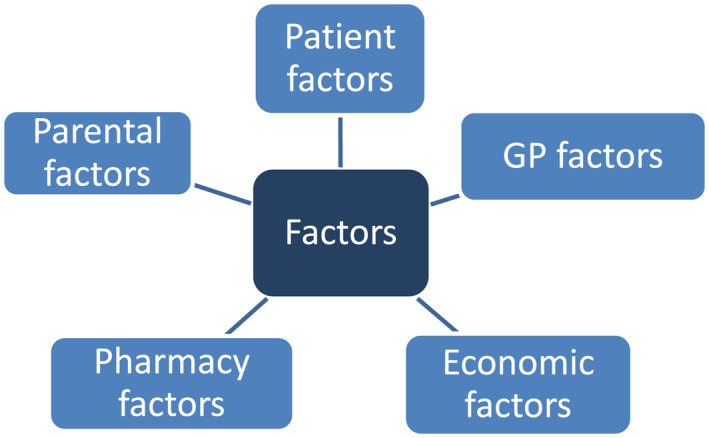
Factors influencing the topical corticosteroids (TCS) treatment strategy of general practitioners (GPs)

According to our participants, several factors played a role in prescribing TCS: patient factors, parent factors, GP factors, pharmacy factors and economic factors (see Figure 1).

#### 4.2.1 Patient factors

As expected, the severity and the number of symptoms played an important role for all GPs in whether they prescribed TCS and what kind of TCS they prescribed. Severity of AD was one factor. If the AD was more severe, all GPs were tempted to start directly with a low‐potency TCS in addition to the emollients or to even to start with a more potent TCS. The age of the child was another factor taken into account when prescribing TCS. One GP indicated never prescribing a TCS for children under the age of four.

Quote 3 (GP 14):

Interviewer: “OK, so the basis is the emollients. But given that they’ve often already been using that or are already familiar with it, you often start straight away with a corticosteroid, right?” GP: “Yes, yes. And if they… I prefer not to do that with really young children, so I don’t like to do it if they’re younger than four. Um… what I do then is I often refer them to the dermatologist to perhaps start with tacrolimus.”

Another GP said they never prescribed a potent TCS to children under the age of 10 because they felt it was never necessary. However, one GP always started with a very potent TCS no matter how old the child was. Other GPs expressed not wanting to prescribe a potent TCS or higher to young children.

The Dutch GP‐guideline suggests to base the potency of TCS on the severity of the AD, the effect and the frequency of exacerbations and take into account the patient's previous experience. Furthermore, a potent TCS for children under the age of 2 years is suggested for short‐term only when AD is severe.

Quote 4 (GP 13):

GP: “In the past, I always used mild TCS. But since, um… [professor of child dermatology] has been working here in the outpatient clinic and he goes for a brief treatment and a very potent TCS. So it’s a very potent TCS from the start.”

Interviewer: “Right, so you do that too…?” GP: “Yes.” Interviewer: “Right, so do you do that with all children, or…?” GP: “I do that with all children as a rule.” Interviewer: “With all children, OK. So the age or whatever doesn’t matter…?”GP: “No.”

#### 4.2.2 Parental factors

It was stated that GPs still notice concerns about the use of TCS among parents. In most cases an extended explanation could reassure parents. However, to reduce corticophobia, two GPs occasionally started with a more potent TCS to get a rapid response and convince worried parents about the efficacy of TCS.

Quote 5 (GP 5):

Interviewer: “Does parental anxiety still play a role? So you start with, um…”

GP: “[…] No… well, sometimes it does of course if it’s quite a severe case. So, um, um… then I might move on more quickly to a slightly tougher treatment. If they’re applying a TCS anyway, it’s often not that clear for the parents whether it’s a mild, moderate or potent. That’s to persuade them that it genuinely works.”

On the other hand, some of the GPs reported postponing a treatment with TCS or not prescribing TCS as a result of fear among parents.

#### 4.2.3 General practitioner factors

Years of experience as a GP seemed to play a role in prescribing TCS. Seven out of eight GPs with more than 20 years of experience prescribed TCS to children when they believed it was necessary, without any doubts.

Quote 6 (GP 8):

GP: “I think that I, um… well, look, it all depends to a certain extent but if it’s not that severe I just start with hydrocortisone butyrate, so that’s a moderate TCS. But if it’s really severe then I’m quite happy to give Dermovate (very potent TCS) for a few days, yes.”

In contrast, the younger GPs with less than 5 years of experience seemed more cautious in prescribing TCS to children; they hardly ever prescribed a potent TCS.

Quote 7 (GP 3):

Interviewer: “If you, um… so don't get a result with a moderate TCS, then you're saying that on rare occasions you will prescribe a potent TCS…””

GP: “Well, I’d have to look at the case more closely…”

Interviewer: “OK, so it’s not something that is in your protocol?”

GP: “No. If that’s the case I’m looking at then I would definitely have to have a good look at the guideline and see right, that’s where I am now, what does the eczema look like, right. I don’t think I’ve ever… if I had to consider that, I’d be thinking what should I do, should I refer them or continue applying the ointment or… well, then I just would have to investigate things a bit further.”

Furthermore, GPs claimed they had no fears about TCS, some were reluctant to prescribe a (potent) TCS, especially in younger children. Eventually it was mentioned that this cautiousness was taught during their medical and GP training, some said that they were afraid of side effects and or that they have limited experience with potent TCS in children. It was stated that GPs had observed side effects from TCS, also in children, which all developed after uncontrolled long‐term use. Hypopigmentation, stretch marks, skin thinning and telangiectasia were named side effects by the GPs.

Quote 8 (GP 11):

GP: “Well for a long time, when we were training and even before that, they always said hormone cream is really bad for you. That your skin will get thinner every time you apply it. Um… you know and that at some point you skin will get damaged, and that it will never recover. [..] Um.. so that’s kind of funny because in the back of my mind I still hear my professor saying that in lectures and you think, oh right shoot.”

#### 4.2.4 Pharmacy and economic factors

Divergent advice about TCS given by a dispensing pharmacist (or assistant) interfered with the advice of the GP and this reinforced the concerns of patients and parents. Overall, this may lead to poor adherence.

Quote 9 (GP 9):

GP: “So I think yes, the advice the pharmacy gives also has big consequences for how people deal with it. I mean, it’s all very well me telling them you should just apply those corticosteroids but if the pharmacist’s assistant then tells them to watch out because it’ll make the skin thinner, well they will simply forget what I told them.”

Additionally, the reimbursement of healthcare insurances can affect a GP's choice of treatment. A few GPs mentioned that in some cases they had to prescribe other emollients or TCS because the one preferred by them was no longer covered by the insurance.

### 4.3 Information given about TCS

The recommended duration of TCS treatment varied among the GPs from a maximum of 5 days to a duration of up to 4 weeks. The rationale behind the recommended maximum duration varied between minimising the risks of side effects and the need to step up therapy when there is no effect. The Dutch GP‐guideline recommends to evaluate the effect of the treatment in one to 2 weeks and recommends to limit daily use of potent TCS till 2–3 weeks due to the risk of local side effects.

Quote 10 (GP 14):

GP: “In terms of duration, I generally say um… for the, um… mild TCS, no longer than a week and after that it should definitely not be continued for a few days. Um… and for the moderate TCS and stronger, um… I usually go for five days.”

Furthermore, the recommendation to apply the TCS thinly was explained by some GPs. The fingertip unit as an indication for the use of TCS and recommend by the Dutch GP‐guideline was not reported by the GPs. The majority of the GPs reported advising apply TCS twice a day, which is in line with the current Dutch GP‐guideline. A few told to apply TCS a maximum of once a day on delicate skin for example, eyelids.

### 4.4 Potent TCS in children

In general, it was remarked that GPs did not have difficulties with starting with a potent TCS in children. However, it is stated that only a potent TCS is prescribed in children above a certain age and/or only in certain areas (i.e. elbows and knees) and in the case of severe AD. Furthermore, overtreatment with TCS and reduced use of emollients were mentioned as a counterargument for not prescribing a potent TCS. It was remarked GPs were concerned patients would use a potent TCS for too long with side effects as a result. GPs also mentioned mild and moderate TCS to be sufficient and said there was no need to prescribe a potent TCS.

It was stated GPs said they occasionally prescribed a potent TCS as the initial therapy in children and had positive experiences with this treatment strategy.

Quote 11 (GP 11):

GP: “So I have no problem with the step‐down approach but it shouldn’t be um… a sort of easy way out, like we say oh, we don’t feel like putting a lot of time into it so we’ll just slap on these kill or cure remedies you know, because in particular with Vaseline if you apply it properly then in many cases of eczema you’re actually fine. So it’s a bit sort of, I’m not against it but I feel you should also continue to consider and assess things carefully, you know.”

## 5 DISCUSSION

### 5.1 Summary

The semi‐structured interviews with GPs in the Netherlands explored the treatment goals, explanations and advice given by GPs about AD in children. Our study revealed multiple factors that play a role in prescribing TCS in children with AD, and that different GPs have different approaches to prescribing potent TCS in children with AD.

GPs said the treatment goal with respect to the disease is mostly focussed on the short term to get the child free of symptoms and itching. About the explanation of the condition, the GPs reported to be more focussed more on the chronic character and the fact that it flares up. GPs told they give advice about the importance of emollients and frequency of use, but not on the amount needed for effective use.

We found that several factors played a role in prescribing TCS. These factors can be categorised into patient, parent, GP, pharmacy and economic factors (Figure 1). All of these factors can influence the treatment strategy of the GPs in terms of whether they start with TCS and if so, which potency group. GPs differ in their attitudes towards TCS: some are cautious about prescribing TCS in (young) children and others, especially GPs with more work experience, are willing to prescribe a potent TCS as an initial therapy. Antihistamines and bath oil are frequently mentioned as additional therapies. Therapeutic garments are prescribed in some cases. GPs with more dermatological experience sometimes prescribe tacrolimus.

### 5.2 Strengths and limitations

As far as we know, this is the first qualitative study to explore what GPs explain and advise about childhood eczema, and which factors influence the GPs' treatment strategy. In contrast to quantitative studies, this qualitative study enabled us to determine why GPs made specific decisions in their management of AD in children.

An adequate sample size was realised as saturation was achieved for the main themes and subthemes. The study was carried out among GPs from two regions in the southern and western part of the Netherlands. This is a wide area with urban and rural settings, and generalisability to other parts of the country is not expected to be a relevant limitation. A limitation of this study is that we do not know if what the GPs say they do is what they actually do in practice. On the other hand, the GPs said this was what they actually do in practice and their answers were not always in line with the Dutch GP guideline, so they were not just giving socially desirable answers.

The first few interviews were conducted by one researcher, who is a GP trainee (Karlijn F. van Halewijn), while all other interviews by a second researcher (Tessa Warendorff) who was finishing her Master's degree in medicine. Research shows that respondents' perceptions of the interviewer can influence the interview interactions.^17^ GPs might have given less extended answers or explanations when the Master's student was conducting the interview. However, both researchers (Karlijn van Halewijn and Tessa Warendorff) attended all the interviews together, so if something was not clear enough the GP trainee explained the questions in greater detail. Potentially, it could inhibit the interviewed when two interviewers were represent. On the other hand, the second interviewer could enrich the interviews and broaden the scope.

### 5.3 Comparison with existing literature

The treatment goal of the GPs was mostly focussed on the short term (relieving symptoms) or educating the patients in adequate self‐management. However, the treatment goal in the Dutch GP guideline is complete remission of the AD.^6^ Knowledge of the recommended treatment goal seems to be lacking, despite AD being a common condition in young children who come to the GP surgery.^18^ In line with above, Powell et al. identified several areas of divergent views between parents and GPs regarding AD. One of them is that clinicians were focussed on just ‘managing’ the condition and parents looked for a ‘cure’ for AD, often fixated on the role of allergy.^19^

Santer et al. found that parents who received more information about AD and explanation of the rationale for prescribing an emollient appeared more convinced of the need for long‐term emollient use to prevent flare‐up.^20^ It seems that this is covered by the explanation the GPs gave in our study. All GPs highlighted the use of emollients as the basis of treatment and the need to apply the emollients generously; however the amount to be used is not discussed.

We did find that the GPs emphasised the chronic character to the patients, but on the other hand they offered the perspective that in most children AD can improve and disappear as they grow older. The discrepancy between a chronic condition in childhood and automatic recovery as they grow older is difficult to understand for parents/patients and may lead to confusion about the course of AD and the treatment strategy. Taesdale et al. found treatment adherence could be supported by stressing that AD is a long‐term episodic condition.^21^ The perception that AD is not considered as a long‐term condition by parents of children with AD is also highlighted by the review of qualitative studies of Teasdale et al. This had implications for the perceived necessity of long‐term treatment. Furthermore this was linked with frustration by patients and carers at the perceived ‘simplicity’ of AD management in targeting symptom control rather than underlying causes.^22^ Additionally, caution is advised when discussing the prognosis for AD with patients and carers, since AD will not always disappear in all children as they grow older. GPs should consider putting less emphasis on a possible improvement and disappearance with age.

It is notable that GPs prescribe medication that is not recommended by the guideline such as very potent TCS and tacrolimus. In the case of antihistamines, evidence that they reduce itching is lacking.^6^, ^23^ Although GPs may have experience with the medication that is not indicated by the guideline, caution is advised. GPs say they advise other therapies like bath oil as suggested by the Dutch GP‐guideline. No evidence of clinical benefit from including bath additives in the management of AD in children was found by Santer et al.^24^

Our results regarding prescribing TCS are partially in line with previous studies, which have found that GPs experience uncertainty about prescription quantities and the use of potent TCS for children and adopt trial‐and‐error approaches to emollient use.^13^ Previous work by Lambrechts et al. showed there is considerable corticophobia among healthcare professionals, especially among pharmacists and GPs.^25^ In our study too there were GPs who were reluctant to prescribe very potent TCS in children. The reason mentioned most often for this reluctance is the ‘fear’ of uncontrolled long‐term use of strong TCS.

Farrugia et al. found that there is a need for re‐education of pharmacists and GPs on the safety of TCS use and the potential impact of their counselling of patients and parents on treatment adherence.^26^ Smith et al. found that targeted, evidence‐based education delivered by a dermatologist caused a major attitude shift to the use and safety of TCS in paediatric AD in Australian pharmacists.^27^

Re‐education may also be needed for Dutch GPs and pharmacist (assistants) to give them more background information on prescribing TCS when needed for the treatment of AD in children. This is desirable not just for GPs who are afraid of using strong TCS in children but also for GPs who prescribe a very potent TCS in every case.

### 5.4 Implications for practice and research

GPs should put more emphasis on the long‐term goal in the treatment of AD. The focus seems to be too much on the short‐term relief of symptoms. Parents and children benefit from a clear explanation of the chronic character of the condition, and its possible disappearance with age should be explained cautiously. More attention and education about the use and safety of TCS in children during the GP training, continuous medical education, and a better implementation of the guideline may improve the consistency among GPs in their advice and prescribing of TCS. Pharmacists (assistants) should not be forgotten in the education about the use and safety of TCS in children. More knowledge and consistent information spread by the primary care team about the correct use of TCS can lead to better education of patients and/or parents, which may lead to better self‐management in children with AD.

## AUTHOR CONTRIBUTIONS

**Karlijn F. van Halewijn**: Conceptualization (equal); Data curation (equal); Formal analysis (equal); Methodology (equal); Project administration (equal); Writing – original draft (equal); Writing – review & editing (equal). **Tessa Warendorff**: Data curation (equal); Formal analysis (equal); Writing – original draft (equal). **Arthur M. Bohnen**: Conceptualization (equal); Methodology (equal); Writing – review & editing (equal). **Mario Veen**: Methodology (equal); Supervision (equal); Writing – review & editing (equal). **Suzanne G. M. A. Pasmans**: Conceptualization (equal); Writing – review & editing (equal). **Patrick J. E. Bindels**: Conceptualization (equal); Writing – review & editing (equal). **Gijs Elshout**: Conceptualization (equal); Formal analysis (equal); Investigation (equal); Methodology (equal); Writing – original draft (equal); Writing – review & editing (equal).

## CONFLICT OF INTEREST

All authors state no conflicts of interest for this article.

## ETHICS STATEMENT

All GPs received written information and provided written informed consent. Data were analysed and stored anonymously. The study was approved by the Medical Ethics committee of the Erasmus Medical Center, Rotterdam (MEC‐2019‐0626).

## Supporting information

Supporting Information S1

Supporting Information S2

## Data Availability

The data that support the findings of this study are available on request from the corresponding author. The data are not publicly available due to privacy or ethical restrictions.
