# Supplementary material for: General practitioners' explanation and advice on childhood eczema and factors influencing their treatment strategy: A qualitative study
Source: Skin Health Dis. 2022 Jul 6;2(3):e147. doi: 10.1002/ski2.147 (PMC9435457; doi:10.1002/ski2.147)
Supplement: Supplementary file 1 — Supporting Information S1 [file SKI2-2-e147-s002.docx]

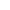
**Appendix 1.
Topic list for interviews with GPs – treating eczema in children**Introductory question: What do you prescribe for the treatment of eczema in children?

General:

Ask for more information using open questions:

- Could you clarify that further?

- Could you give an example?

– Please explain...

– Do you always take this approach?

What do you explain about eczema?

What do you aim for with your treatment?

When do you prescribe emollients?
 Which emollients do you prescribe and why?

Why do you prefer a cream or ointment?

What advice do you give when prescribing emollients?

Do you sometimes recommend or prescribe bath oil? Why do/don’t you do that?
 TCS and other medication

What medicines treatments do you prescribe for eczema?

What determines whether you prescribe a topical corticosteroid (TCS)?

What class of TCS do you choose as an initial treatment?

Do you choose a cream or an ointment, and why?

What does that depend on? (Severity, age, size of affected area, parental anxiety?)
 Does the parent’s anxiety play a role?

What information do you give about the medication (for example, on the duration of the treatment) and why?
 Are you afraid of side effects of a TCS? If so, which side effects and why?

Apart from emollients and TCS, do you prescribe other medication for the treatment of eczema?

Other aspects:

What other recommendations do you give to children with eczema and their parents?
 What do you do if parents suspect a food allergy?
 What advice do you give about maintenance therapy?

What advice do you give about showering and bathing?

What advice do you give about phasing out the treatment? Do you provide a phasing-out schedule?
 What agreements do you make about follow-up? Do you make check-up appointments?
 What are reasons for referring a child? Who do you refer them to?

Potent topical corticosteroid:
 Would you prescribe a potent TCS for a child with moderate eczema as an initial treatment?

What could be reasons not to?

General information about the GP
Age:
Sex:
Practice size:
Years of experience:

Dermatological interest: professional experience with dermatology/child with eczema/personally has or had eczema
Affiliated to an academy? Yes/no (trainer, research, primary GP, specialist GP)
