# Supplementary material for: General practitioners' explanation and advice on childhood eczema and factors influencing their treatment strategy: A qualitative study
Source: Skin Health Dis. 2022 Jul 6;2(3):e147. doi: 10.1002/ski2.147 (PMC9435457; doi:10.1002/ski2.147)
Supplement: Supplementary file 2 — Supporting Information S2 [file SKI2-2-e147-s001.docx]

**Appendix 2.**
**Main and sub themes:**

1. General advice main themes:
   - Lifestyle advice
   - Bath oil
   - Bath and shower
2. Influencing factors main themes:

- Patient factors
- Parent factors
- Doctor factors
- Pharmacy
- Economic factors

Subthemes in blue
